# Adapting a social network intervention for use in secondary mental health services using a collaborative approach with service users, carers/supporters and health professionals in the United Kingdom

**DOI:** 10.1186/s12913-022-08521-1

**Published:** 2022-09-09

**Authors:** Helen Brooks, Angela Devereux-Fitzgerald, Laura Richmond, Neil Caton, Alice Newton, James Downs, Karina Lovell, Penny Bee, Mary Gemma Cherry, Bridget Young, Ivaylo Vassilev, Clare Rotheram, Anne Rogers

**Affiliations:** 1 Mental Health Research Group, Jean McFarlane Building, Division of Nursing, Midwifery and Social Work, School of Health Sciences, Faculty of Biology, Medicine and Health, University of Manchester, Manchester Academic Health Science Centre, Manchester, M13 9PL UK; 2 Patient and Public Involvement Contributor, University of Manchester, Manchester, UK; 3 Greater Manchester Mental Health NHS Foundation Trust, Manchester, UK; 4 Department of Primary Care and Mental Health, Institute of Population Health, University of Liverpool, Liverpool, UK; 5 Linda McCartney Centre, Liverpool University Hospitals NHS Foundation Trust, Prescot St, Liverpool, UK; 6 Department of Public Health, Policy and Systems, Institute of Population Health, University of Liverpool, Liverpool, UK; 7 Faculty of Health Sciences, University of Southampton, Southampton, UK; 8 The Life Rooms, Merseycare Foundation Trust, Liverpool, UK

**Keywords:** Social networks, Mental health, Co-adaptation, Patient and public involvement, Implementation

## Abstract

**Background:**

Social integration, shared decision-making and personalised care are key elements of mental health and social care policy. Although these elements have been shown to improve service user and service-level outcomes, their translation into practice has been inconsistent and social isolation amongst service users persists.

**Aim:**

To co-adapt, with service users, carers/supporters and health professionals, a web-based social network intervention, GENIE™, for use in secondary mental health services. The intervention is designed to support social activity and preference discussions between mental healthcare professionals and service users as a means of connecting individuals to local resources.

**Methods:**

In Phase 1 (LEARN), we completed two systematic reviews to synthesise the existing evidence relating to the i) effectiveness and ii) the implementation of social network interventions for people with mental health difficulties. We undertook semi-structured interviews with a convenience sample of 15 stakeholders previously involved in the implementation of the intervention in physical healthcare settings. Interviews were also conducted with 5 national key stakeholders in mental health (e.g., policy makers, commissioners, third sector leads) to explore wider implementation issues.

In Phase 2 (ADAPT), we worked iteratively with eight service users, nine carers, six professionals/volunteers and our patient and public advisory group. We drew on a framework for experience-based co-design, consisting of a series of stakeholder consultation events, to discuss the use of the social network intervention, in mental health services. Participants also considered factors that could serve as enablers, barriers, and challenges to local implementation.

**Results:**

Across the stakeholder groups there was broad agreement that the social network intervention had potential to be useful within mental health services. In terms of appropriate and effective implementation, such an intervention was predicted to work best within the care planning process, on discharge from hospital and within early intervention services. There were indications that the social connection mapping and needs assessment components were of most value and feasible to implement which points to the potential utility of a simplified version compared to the one used in this study. The training provided to facilitators was considered to be more important than their profession and there were indications that service users should be offered the opportunity to invite a carer, friend, or family member to join them in the intervention.

**Conclusion:**

The GENIE™ intervention has been co-adapted for use in mental health services and a plan for optimal implementation has been co-produced. The next phase of the programme of work is to design and implement a randomised controlled trial to evaluate clinical and cost effectiveness of a simplified version of the intervention.

**Supplementary Information:**

The online version contains supplementary material available at 10.1186/s12913-022-08521-1.

## Background

Mental health difficulties are highly prevalent with long-lasting impacts. Approximately 2.8 million people were in contact with adult mental health and social care services in the UK in 2020/2021 [1]. There are more disability-adjusted life years lost per year to mental health difficulties than any other health condition in the UK, including cancer and heart disease [2].

Involving service users in decision-making around their care, offering treatment choices and promoting recovery are central aspirations of contemporary mental health and social care policy initiatives and quality improvement guidance [3]. The 5-year plan by the Mental Health Taskforce is an example in a series of government mandates advocating for service user and carer focused interventions [4, 5]. This upholds shared decision-making and personalised care as guiding principles of mental health service design and delivery [3]. In direct response to these reports, the Government has pledged to provide more personalised and integrated care to people experiencing severe and/or enduring mental health difficulties [6].

Social networks constitute a set of connections and ties, linking people to relationships and resources which can support them to manage their mental health. Social networks have been found to positively influence recovery for people with severe and/or enduring mental health difficulties [7]. Social connectivity and valued activities have been found to be important in recovering and living everyday life with a mental health problem [8]. However, it has also been demonstrated that people with severe and/or enduring mental health difficulties have smaller social networks of poorer quality [9–11]. Therefore, enabling patient activation and improving social integration are necessary components of providing community-based support for people with mental health difficulties. Recent evidence for this population concluded that social networks mediate the effects of social isolation and loneliness and enhance self-management [7, 12, 13]. Furthermore, social relationships have been identified as key mechanisms for behaviour change in strengths-based mental health and social care interventions [14].

Social network interventions which connect people to meaningful and valued activities in domestic and local environments can improve the size and quality of social networks and their collective efficacy [15, 16]. In doing so, they extend access to sources of support that can help to both prevent and manage mental health difficulties [17]. This approach has been shown to be successful for a range of long-term physical health conditions [18, 19] but has been slow to translate to mental health care [20]. However, social connectedness to assist recovery requires navigation, eliciting preferences and engagement by both individuals seeking assistance and those involved with mental health work designated with a supportive and therapeutic role [21].

Social network interventions offer the advantages of strengths-based perspectives. These are approaches which emphasise the mapping of assets individuals have at their disposal (e.g. in the community) as a means of engaging people to talk about their social situation and community [22]. In turn in a mental health service setting these can open up new conversations and dialogue about personal experience and everyday living. This fits with essential elements of the recovery processes identified by Leamy et al. (2011) of: connectedness; hope and optimism about the future; identity; meaning in life; and empowerment and collectivist notions of recovery [23].

### A social network intervention

GENIE™ provides an exemplar of an evidence-based networking tool designed to help people identify and reflect on existing networks as a starting point from which to extend and suggest further connection and engagement with local and online resources. It also helps people to extend networks by facilitating resource navigation and linking people to additional resources (people, social activities, pets, things) and thus to support that individuals experience as valuable, acceptable and supportive [24].

It comprises three steps: a) network-mapping and visualisation for reflexive engagement b) identification of gaps and preferences for social support and engagement, and c) facilitated navigation of online and offline resources based on individual preferences, and involvement of other social network ties.

Mapping social networks provides professionals and service users with a visual representation and means of thinking about the quality of contact with others and social activities that people currently have. It brings into focus what other network engagement may be available and accessible to them. Users receive visual feedback through a pictorial map of their network. Finally, the preferences for new activities are elicited and discussed in the context of information on local community resources to attend or engage with possibly with the help of existing network members to facilitate engagement (via Google maps) (Additional file 1: Appendix 1).

GENIE™ has an existing evidence base to support its utility for adults living with a range of long-term physical health conditions (e.g. chronic obstructive pulmonary disease, diabetes, and early stage chronic kidney disease) which demonstrates a positive impact on some mental and physical health indicators, quality of life and relationships, and connection to the local community with potential for cost containment within the NHS [19, 25]. In terms of feasibility and usability, previous research suggests that in using this tool clinicians strengthened their understanding of patients’ personal social networks and needs, and patients reported less social isolation [26]. Its use has shown to be enhanced when supported by volunteers to expand patients’ social networks and link them to relevant health and social care resources [26]. The intervention operates through the disruption and reconstruction of social networks, challenging/supportive facilitation and change and reflection over time concerning network support. Visualisation of the network enables people to mobilise support and engage in new activities. The tool works best when it is aligned synergistically to a facilitators’ role of linking people to local resources [24].

However, this social network intervention has not yet been used within mental health services. Given the relevance of recognising the impact of context on the workability of social network interventions, the potential differences in the experience, trajectory and marginalisation of mental health conditions compared with physical health conditions [27], the intervention needs to be robustly explored and adapted for use in mental health services.

Our study, The Co-Adaptation of a Social Network Intervention to Support Recovery for People living with Severe Mental Illness (ConNEct) aimed to co-adapt, a web-based social network intervention which was designed to support discussions between mental healthcare professionals and service users about their care and connect these individuals to local resources.

### Objectives

1. To develop an in-depth understanding of the potential role of a social network intervention (GENIE™) within mental health services, from the perspective of service users, carers/supporters, and mental health professionals.
2. To identify the individual and organisational barriers and facilitators to the use of social network interventions in mental health settings.
3. To co-adapt with service users, carers/supporters and professionals a social network intervention for testing and evaluation in secondary mental health services.
4. To develop a model of implementation, including specific determinants of success informed by recent evidence and implementation theory and the perceptions of service users, clinicians, and other key stakeholders.

### Patient and public involvement (PPI)

PPI has been an essential component of the current study and has included the recruitment of a co-applicant and researcher with lived experience of severe and/or enduring mental health difficulties, a PPI advisory group and consultation workshops with mental health service users, carers/supporters, and clinicians/volunteers. The advisory group met quarterly, and its role was to provide advice and guidance to the research team drawn from lived experience of mental health difficulties and secondary care services, in a variety of ways including the development of research materials and recruitment processes, developing patient-mediated materials, contributing to the systematic review, inputting into data analysis, co-authoring publications and supporting the dissemination of study findings.

## Methods

The study was informed by the Medical Research Council’s Framework for Developing and Evaluating Complex Interventions [28, 29] and comprised two distinct but interrelated phases: Phase 1 LEARN and Phase 2 ADAPT.

### Phase 1: LEARN

Phase 1 involved undertaking a needs assessment which included two systematic reviews and qualitative interviews with people who have previously used GENIE™ in physical health and community settings and those involved nationally in mental health policy and practice.

#### Systematic reviews

Two systematic reviews were undertaken to provide an up-to-date synthesis of evidence relating to the effectiveness (review 1) and implementation (review 2) of social network interventions for people with mental health difficulties.

##### Review questions

Review 1:

- What is the effectiveness of interventions designed to improve the quantity and/or quality of social networks of adults with mental health difficulties?
- What are the factors that influence the effectiveness of social network interventions for people with mental health difficulties?

Review 2:

- For people with mental health difficulties, what social network interventions work best, for whom and in what contexts?
- What are the barriers to and facilitators of the implementation of social network interventions for people with diagnosed mental health conditions?

##### Methods

Review 1: Articles were eligible for inclusion if they reported data from randomised controlled trials (RCTs) on the effectiveness of interventions designed to improve social networks for adults (18+) with mental health difficulties. Papers were independently reviewed for inclusion with conflicts resolved through consensus. Included papers were quality-assessed, relevant data were extracted, and synthesised narratively. Risk of bias was assessed using the Cochrane Risk of Bias Tool [30]. Due to the heterogeneity of included studies, it was not possible to undertake a meta-analysis and a narrative synthesis was conducted.

Review 2: Journal articles or dissertations with primary data on the implementation of interventions designed specifically to improve and/or measure social network quantity and/or quality for adults (18+) with mental health difficulties were included in this review. All types of study design were included. Papers were independently reviewed for inclusion with conflicts resolved through consensus. Included papers were quality-assessed, data-extracted, and synthesised narratively. Analysis had a specific focus on identifying the relevant mechanisms and how they work in different contexts, with the aim of developing a deeper understanding and to allow theory-building. The Mixed Methods Appraisal Tool (MMAT) [31] was used to assess the quality of the included papers as it was devised for a broad range of research, as arose in this review.

Further detail on the methods and results of these systematic reviews are available separately [20]. Review 1: https://www.crd.york.ac.uk/prospero/display_record.php?RecordID=206490. Review 2: https://www.crd.york.ac.uk/prospero/display_record.php?RecordID=206423.

#### Qualitative interviews

##### Design

A qualitative, exploratory design was used to elicit current understanding of the design and use of the intervention in mental health services. We undertook semi-structured interviews with multiple stakeholders who have previously used GENIE™ across a range of service settings. Such methods are recommended to fully understand the complexities and nuances of potential implementation [29]. Interviews were also undertaken with national key stakeholders involved in social networks and mental health (e.g. policy makers, commissioners, third sector leads and additional key influencers) about wider implementation issues [32].

##### Participants

A database of potential participants for both the previous users of GENIE™ and the national key stakeholders was compiled by the study team and this was combined with the use of snowballing sampling techniques [33], whereby interviewees were asked to nominate other people who may also meet inclusion criteria.

Potential participants received a direct invitation by email from the study team which included a study participant information sheet. Participants had the opportunity to ask questions before a mutually convenient time and date for the interview was arranged. Of the 21 potential participants who were identified as previously using GENIE™, 15 provided informed consent and took part in an interview. During the process of data collection, it became apparent that one participant did not meet inclusion criteria as they had no direct experience of using the GENIE™ intervention and as a result their data were not included in the analysis. Of the 10 national key stakeholders who were approached, 5 took part in the study. Reasons for non-participation included not being contactable, being on maternity leave, not meeting eligibility criteria, and not having enough time. 15 previous users of GENIE™ and 5 national key stakeholders consented to take part in the study. For more information relating to phase 1 study participants, see Table 1.

**Table 1:** Demographic information on phase 1 participants

	Previous GENIE™ users (***n*** = 15)^a^	National stakeholders (***n*** = 5)^b^
**Gender**		
Male	3	3
Female	11	2
**Age**		
35–39	4	1
40–44	1	
45–49	1	3
50–54	2	
55–59	2	
60–64	3	
65–69	1	
**Ethnicity**		
White British	8	3
White Other	4	1
White Unspecified	2	
**Professional role**		
Researcher	7	
Project Manager	1	
Academic	3	
NHS Senior Manager	1	
Clinical Academic	1	1
Social Worker	1	
Professor		2
Third Sector Lead		2
**Time in role**		
< 1 Year	2	
1–4 years	5	
5–9 years	2	2
10–14 years		1
15–19 years		
20–24 years	1	
25–29 years		1
30–34 years		
35–40 years	1	
41–45 years	1	
Retired	1	
Unspecified	1	

^a^Demographic data presented for 14 participants as one participant did not meet the inclusion criteria

^b^One demographic form not returned

For the previous users of GENIE™, data saturation was thought to have occurred after 12 interviews and an additional 3 were undertaken to ensure further data collection did not meaningfully add to the results. For the national key stakeholders, each participant was expected to have unique specialist knowledge, therefore we were not expecting saturation in these interviews.

##### Data collection

One-to-one interviews were undertaken by ADF and LR (research associate and lived experience research assistant who were both female and had qualitative research experience) via telephone. No participants were known to data collectors prior to involvement in the study. Interview schedules were informed by the Consolidated Framework for Implementation Research (CFIR) [34] which emphasises the importance of evaluating complex interventions, including those with an element of online delivery, both within existing care contexts and broader social and organisational contexts [34]. CFIR provides a standardised list of constructs compiled from previous studies to support the identification of factors that are most salient to the implementation of a particular intervention [34]. This is a model which comprises a taxonomy of 39 operationally defined constructs across five domains that influence the implementation of complex interventions. The domains relate to the planned intervention, the contexts in which the implementation activities will occur (the ‘inner’ and ‘outer’ settings), the individuals involved, and the process of intervention delivery. This was designed to orient us to the nature of work required at multiple levels within a system for GENIE™ to be successfully implemented and its potential for scalability. Interview schedules were also reviewed by the patient and public advisory group to ensure their meaningfulness and relevance to the project aims overall. Please see Additional file 2: Appendix 2 for example interview questions.

##### Data analysis

Interviews were transcribed verbatim and anonymised before being analysed using Framework Analysis [35] which allowed for both inductive and deductive coding. Analysis of data drew on the constant comparative method [36].

Deductive coding was informed by the CFIR [34] and inductive analysis considered any data that fell outside this framework, ensuring that no important data was excluded. Analysis followed the seven stages of framework analysis [35]:

- 1: Transcription: Interviews were transcribed verbatim by an external transcription company.
- 2: Familiarisation with the data: Transcripts were first read and re-read by members of the research team in order for analysts to familiarise themselves with the data.
- 3. Coding: Each transcript was coded independently by two of eight members of the research team [35]. An analyst with lived experience of mental health difficulties undertook one set of coding for each of the transcripts.
- 4. Developing an analytical framework: Inductive codes were mapped to CFIR components with duplicates and redundant codes removed. No codes were identified that could not be incorporated within the CFIR framework. Codes were compared within and across cases, paying particular attention to negative cases and any reasons for identified discrepancies.
- 5. Applying the analytical framework: Transcripts were revisited as a whole to index the full set of transcripts to the CFIR domains.
- 6. Charting into the framework matrix: An excel spreadsheet was used to generate a matrix using CFIR domains and chart data into the matrix. This stage of the process involved summarising data relating to each CFIR domain for each transcript. Illustrative quotes were linked to summaries for purposes of illustration.
- 7. Interpreting the data: The final stage of the process involved comparing summaries between transcripts to develop interpretations of included data within each CFIR domain. This resulted in descriptive accounts of the potential implementation barriers and facilitators being developed for each of the five components. This process was undertaken collaboratively with analysts with lived experience of mental health difficulties and interpretations were checked with members of the research team with experience of providing services within secondary mental health care settings.

##### Trustworthiness of data analysis

We employed a number of strategies to ensure trustworthiness of data. These included checking 50% of transcripts against the original audio-files to ensure data accuracy prior to analysis, having two members of the research team independently code each transcript, having regular meetings between coders and the wider research team and keeping a documented audit trail of codes, frameworks and matrices. In line with the recommendations for framework analysis, we also required all coders to develop and document memos to support later interpretations and synthesis of data across transcripts.

We endeavoured to ensure that interpretations were grounded in the experience of providing and receiving care in secondary mental health services by ensuring that each transcript was independently coded by a researcher with lived experience of mental health difficulties. We also checked interpretative summaries with two additional members of the research team who had clinical experience of providing care within secondary mental health service settings.

### Phase 2: adaption of the intervention for mental health services

We used a framework for experience-based co-design developed by King’s College London [37] to co-adapt the intervention and co-produce implementation strategies to support the future use of GENIE™ within secondary mental health services.

#### Participants

Participants were invited directly by Merseycare NHS Trust, and adverts were also placed on Merseycare’s social media. Participants were purposively sampled to ensure diversity in terms of age, gender and ethnicity:

- 49 service users expressed an interest in taking part in the study. 12 were invited to attend the focus group, of whom 8 attended (6 for the full event).
- 30 carers/supporters expressed an interest in taking part in the study. Given the attendance rates at the service user focus group, 15 were invited, of whom 9 attended. All participants attended for the full event.
- 12 professionals/volunteers expressed an interest in taking part in the study. All were invited to attend and 5 attended. An additional participant took part in a one-to-one consultation workshop, taking the total number of included professionals to 6. This workshop took place in January 2022, at the height of the Omicron wave in the UK, which is likely to have impacted recruitment.

For more detail on phase 2 participants, see Table 2:

**Table 2:** Demographic information on phase 2 participants

	Service users^a^	Carers	Professionals
**Gender**			
Male	3	7	3
Female	2	2	3
Male-to-female transgender	1		
**Age**			
18–24	1		
25–29		6	2
30–34	1		
35–39		1	
40–44	1		
45–49	1		
50–54			
55–59			
60–64	1	2	2
65–69	1		1
**Ethnicity**			
White British	5	1	4
White Other		1	1
White Unspecified		2	
Mixed	1		
British Asian		1	1
African American		3	
Black Unspecified		1	
**Professional role**			
NHS service team leader	N/A	N/A	1
Psychiatrist			1
Social worker			1
Support Worker			2
Spiritual/pastoral care volunteer			1

^a^Based on participants who attended the full event

#### Data collection

##### First consultation workshops

Information about the intervention and findings from phase 1 of ConNEct were presented to three stakeholder groups: 8 service users, 6 professionals/volunteers and 9 carers/supporters. Workshops were designed so that stakeholders could co-create recommendations for the adaptation and future use of GENIE™ within secondary mental health services and consider any potential barriers and facilitators to implementation.

Workshops lasted between 60 and 90 minutes, were held via Zoom, and were digitally recorded and transcribed verbatim.

##### Sustained smaller team co-design work

The research team worked closely with the patient and public advisory group to develop the implementation strategy from the priorities identified during the consultation workshops. No recommendations were made during the initial workshops for changes to GENIE™ itself as participants deemed this to be unnecessary.

##### Review event

The proposed implementation strategy was presented back to those who participated in the three consultation workshops in February 2022. Participants reviewed and provided feedback on the proposed evaluation protocol and implementation strategy. A revised manual for the proposed intervention is being drafted in collaboration with the patient and public advisory group and programme management group.

#### Analysis

Recommendations were extracted from each workshop transcript and mapped to a table in Word. Similar recommendations were combined with redundant ones removed. Multiple columns were added to the table in order to tabulate consensus between stakeholder groups and identify those recommendations that were endorsed by more than one group (Table 4). The synthesis table was checked by a second member of the team to ensure no data was lost during this process. The resultant table was used to inform the small co-design activities.

During the smaller team co-design activities, the research team in collaboration with the patient and public advisory group reviewed the table compiled from the initial workshops. This was undertaken through discussion which informed by the APPEASE criteria in order to reach consensus about the implementation recommendations to be taken forward [38]. The APPEASE criteria have been designed to support context-based decisions in relation to intervention content and delivery and comprise affordability, practicability, effectiveness and cost effectiveness, acceptability, side effects/safety and equity considerations [38]. Agreement was reached through discussion about which recommendations were considered to meet relevant APPEASE criteria and would therefore be taken for forward in the planning of subsequent studies.

#### Ethical considerations

This study received ethical approval from the Health Research Authority (Ref: 287584) and the North East - York Research Ethics Committee (Ref: 20/NE/0234).

The key ethical issues relating to this study relate to confidentiality, participant anonymity and informed consent to participate. We considered the risk to participants relating to distress during participation to be low given the study activities but designed a distress policy should this be necessary. There were no instances during the study whereby the distress policy needed to be implemented.

Robust data management procedures were implemented to minimise risks to study data being compromised. This included anonymisation of study data at the point of transcription and ensuring any quotes used in dissemination activities could not identify study participants. During phase 2 workshops, we agreed ground rules with attendees which included agreement that all information discussed should be treated as confidential and should not be discussed outside of the group.

Study documentation including information sheets and consent forms were written to current ethical standards and reviewed for clarity by our Patient and Public Advisory group. All participants who took part in the research process provided informed consent to participate in study activities.

## Results

### Phase 1

#### Systematic reviews

##### Review 1

Initial searches returned 22,367 manuscripts for review. Of these 841 were deemed suitable for full-text appraisal and 9 studies, randomising 2226 participants, were included in the review. Four focused on those with a diagnosis of schizophrenia or psychosis, one on major depressive disorder, and four on all mental health diagnoses.

The review determined that extant literature is of unclear quality and the evidence base relating to the use of social network interventions for people with mental health difficulties is in its infancy. However, the analysis provided preliminary evidence that network interventions which focus on connecting to and supporting social activities appear to hold the most promise. Whilst data on cost effectiveness and acceptability were limited, they highlight encouraging potential economic feasibility and acceptability for evaluating these interventions.

The review suggested that further research is required to understand the underlying mechanisms of action and to develop evidence-based recommendations for health services. Future research should i) incorporate nested process evaluations to understand and optimise implementation, ii) ensure meaningful patient and public involvement (PPI) to increase intervention uptake and acceptability, and iii) incorporate high-quality cost data to facilitate economic modelling.

Further detail on the results of this systematic review on the effectiveness of social network interventions for people with mental health problems is available here [20].

##### Review 2

Initial searches returned 22,367 manuscripts for review. Of these 841 were deemed suitable for full-text review and a total of 54 papers were included, consisting of 17 RCTs; 12 other quantitative studies; 14 qualitative studies and 11 mixed methods studies. Studies were included from the following countries: UK 25, USA 8, Australia 5, China 2, India 2, Ireland 2, Italy 2, Netherlands 2, Sweden 2, Canada 2, Denmark 1, Hungary 1. Analysis identified a range of micro, meso and macro level facilitators and barriers to implementing social network interventions for people with mental health difficulties which were sensitised using the CFIR. Exemplar factors are highlighted below:

- Intervention: Accessibility, staff time, lack of training, length of intervention and sustainability, cultural alignment, financial support, links to existing organisations.
- Outer setting: Socio-economic setting, community infrastructure, social hierarchy.
- Inner setting: Place (safety, stigma, understanding of mental health).
- Individual: Time, confidence, health, financial resources, readiness for participation, previous negative experiences, individual personal circumstances, literacy.
- Process: Flexibility, allowing people to go at their own pace depending on circumstances, humour and engagement with valued activities, one-to-one work with facilitators to promote engagement, stigma reduction, structured support for real-world interaction.

Both reviews highlighted a lack of mental health focussed PPI in research in this area and identified a need for enhanced PPI to ensure the future acceptability and uptake of social network interventions.

#### Qualitative interviews

Key findings interpreted from the qualitative interviews are presented under each of the five CFIR components [34] and exemplar quotes can be found in Table 3.

**Table 3:** Phase 1: exemplar quotes

CFIR component	Exemplar quotes
Intervention characteristics	*They just wanted financial impact: how does it help them or how does it stop people coming into the clinic so much and how does it…? You know, they don’t like people not attending clinic when they’re booked in; they don’t like people not talking to them, not coming into clinic, and they don’t like people having complications or needing to go to A&E and things like that. So if you talk about how it impacts on those key outcomes, then they’re interested.****C1011*** *I think just the process itself is just, you know, it gives people a chance to do something that they’ve probably never done. I think to plot someone’s social network map and have that visual of those relationships of the people and the places that are important to them. I think I'd certainly never done that for myself and I think doing that is a nice process for people to be able to do and to see. And to just have that, especially for, we’ve done it with people who are lonely and socially isolated, and to have that time just to be listened to and to speak about your networks and I find that people have really enjoyed the process.***C1005** *You know, draw out issues around relationships with grown up children and that sort of thing. So I think it helps draw out and helps better understand some of the social dynamics, which might be going on. Because it allows you to open up those conversations, as to why sort of someone might not see their daughter and grandchildren any more than six…you know, than twice a year, for example. Even though they don’t live very far away, or…and this sort of thing.***C1013** *Having a resource base, which is up to date more generally, is just a really useful tool. So we found in our work that where teams have resource inventories, they are often out of date or people don't necessarily have the personal relationship with people in the local community groups to really facilitate the warm referrals.****KI1003*** *I think it was the fact that there’s a database attached and it was not always that clear how that was being updated or whose responsibility that would have. So, there was one organisation, and they seemed quite keen initially and then ultimately they said we can't waste, our time is so precious, we can't waste it if the information that people get isn't going to be up to date, then it makes all the rest of it redundant, if you like, you know it's sort of wasting people's time. So, although in theory, it's good, they weren't interested unless there was a guarantee that the end information would be accurate. So, I thought that was quite telling really.***C1007** *If I give them a list of three things, and go, well, oh, look, here’s three things I’ve printed off for you. They can come back next week, well, none of those were any good. You know, have you got some more? And what you’ve done there, you’ve created a passive dependency. Where if a person’s gone out to find information and it hasn’t worked out, they’re much more likely to go, okay, I’ll ask my grandson to come up with some more.***C1013** *It was the network mapping, that’s the most eye-opening bit of it, and then the linking people to resources, I think people quite liked just having that as a portfolio of options, they didn’t necessarily take them up, but I think the actual networking, get people to think differently, that’s what opened up stuff more actually.***C1008**
Outer setting	*Although you can particularly make the argument for people with mental health problems because of the wellbeing gains from social interaction, from physical activity, from well, you name it, nature and so on, all of the things that you find within communities I think have a, not a potentially, not everything has a wellbeing gain but potentially has a positive impact on wellbeing.***KI1003** *So, quite a lot of the time, people are going to community groups with a squillion things on their minds like issues, concerns, troubles and for us, we were finding actually loneliness is a bit of a luxury because I can’t pay my bills, I can’t get my kids to school, I haven’t got any heating, I don’t have any food, also, my mental health’s pretty rubbish.***C1003** *It all really depends on that person’s sort of level of confidence and where they are, and just how big steps they can begin to make, and how quickly towards making changes. You know, for some people, it’s…you know, for someone who’s sort of really struggling to even get out of the house, or get on a bus and all the rest of it, because they’re so fearful. And, you know, got a quite high level of anxiety and moderate depression and what have you, it’s…you know, they’re not going to suddenly go, oh, swimming group, that sounds excellent, I’m off down the swimming pool tomorrow. You know, we know it just doesn’t happen like that.***C1013** *It was…I think it was really difficult if you had someone who was very disabled physically and very…had a tiny, tiny social network. And there were people…we did come across people like that who were really isolated, and you had really struggled to find anyone in their social networks except health professionals***C1008** *So, a lot of the, I guess, response we got from people that we came into contact with at the mosque was that like people in that community aren’t lonely as such or it’s maybe different or people don’t really admit that they’re lonely. So, I think that was an issue…that might be linked to kind of loneliness and stigma of loneliness***C1001** *There were some community organisations that weren’t relevant because they were just supporting people with really, really complex needs and loneliness was essentially a luxury. So, I guess the lesson for us, for me, was* GENIE^TM^*works in a context… you have to understand the users, I think, and understand the people accessing that context.***C1003**
Inner setting	*I think that was the thing that we found was that some of the bigger organisations have the reach but they don’t necessarily…it doesn’t really align with the job role. But then some of the smaller organisations or charities or…you know, like one of the people that we work with a lot are a local organisation which do like befriending and take people on, in ordinary times, not obviously COVID, they do things like tea parties and that sort of stuff and their ethos is around kind of like being neighbourly and this sort of stuff. I don’t know, like, they had the skills but then the problem with some of these organisations, is that they don’t have the resource because they don’t have money and they’re in this like commissioning cycle but they really bought into it. So, it really does depend on the structure of the organisation as well.***C1001** *I mean, you know, the uptake of tools such as* GENIE^TM^*, I think is always sort of slightly problematic. I mean, regardless of the tool, whether it’s* GENIE^TM^*or any approach, you know, sometimes it’s just a lack of timing…sorry, not lack of. Luck of timing, so you get in when everyone’s sort of receptive and everything, so yeah. So I think it does depend on the ethos of the organisation and to what extent they understand the importance of the social side of health outcomes***C1013** *I think what we learnt quite early from them and other organisations similar to them was that actually when we’re having these conversations about identifying participants and then how the study will work, because we sort of run in a pragmatic way, that actually you need the buy-in from the high levels. So in a different organisation, which is the Housing Association, they actually wrote it into people’s objectives for the next three or six months, so that the people working in the study actually had like formalised time to dedicate to the study. But it also had to have, you have to buy-in from all the levels. So maybe depending on the structure of the organisation, high level people need to buy into it, but then also the people who are doing the work on the ground, and their managers.***C1001** *Getting it out into the organisations, it’s getting the training right, getting that enthusiasm right and finding the right people within organisations to actually deliver it and then we’ve got management buy-in, because that seems to be essential with any implementation thing that everybody agrees to it and supports it, so there’s knowledge about that, so that’s sort of what makes things fly***C1008**
Characteristics of individuals	*Or, you know, if they’ve lived somewhere all their life, you know, talk about local parks that they’ve been to and memories and things, so draw out things from their past rather than…because, I wouldn’t want them, me to leave and them just think, oh gosh, I’ve got no one in my map and I feel lonely and make them feel worse than before you’ve got there. So, yeah, that’s how I would try and divert the conversation when people don’t have many people in their network initially.***C1005** *But then like, for example, in one of the local church groups, we essentially found somebody who was like a real champion of community engagement and* GENIE^TM^*and that sort of stuff. And so after we had trained her, we then were able to kind of like direct people from different organisations in towards her, if that makes sense. So she had the capacity, she was retired and she had the capacity to do it.***C1001** *Only in that the champions also seemed to be people who were able to build rapport quite quickly with their clients, and because of that rapport they had an effective way of encouraging the client to then perhaps try out some of the activities that were identified through* GENIE^TM^*.***C1004** *I think peer facilitators are absolutely fantastic. I don’t think we use them enough. And with the right training, the right supervision and the right support, they are quite often, much more effective than professionals. Professionals tend to slip into telling people what to do, providing solutions to people, is kind of TFA. It’s not a criticism, it’s just, you know…it’s back to this, quite often they are working more in a crisis arena as well.*
Process of implementation	*So we went through a system where, when we were approaching different organisations or community groups or whatever it was, obviously, like I said, we went through the whole spectrum, that we would attempt in the beginning to organise a meeting with the team plus all of the different…all of the stakeholders within the organisation, so that we could…the questions could be asked about what the facilitation process looks like, what the recruitment process looks like and that everybody has the chance to ask those questions and hear the answers all together.***C1001** *I think there was definitely more engagement [before the pandemic]. And from my point of view, again I find it easier to remember, I felt I got to know that person a little bit, whereas over the phone I sort of, I don't know them that well or at all and it's harder, because I’ve done follow up calls, I find it harder to recall who they are.***C1007** *But I think, actually, doing the pre-implementation which is around going in and speaking to the organisations, getting an understanding of the work they do, who they are, who they work with, the referral pathways in and out and really sort of understanding their context and finding what little flex there was in the context and then working with* GENIE^TM^*to flex a bit as well and fit that in, that’s where it was really successful.***C1003** *So, two or three facilitators come to mind that really embraced* GENIE^TM^*, but they weren’t afraid of it and they weren’t afraid to sort of make it their own, if you know what I mean, and it seemed to really resonate with what they wanted to offer their clients and how they worked.***C1004** *And I suppose I think coming from very much a community background, I was quite conscious that a lot of it was very academic, you know with a lot of emphasis on the academic side of it and the research side of it. And my feeling was some of those facilitators weren't really interested in that. They just wanted to know: how does it work, what's my role, what do I have to do? And I thought maybe the emphasis should have been more on the practical side of it really and then maybe answering questions, rather than the emphasis being on the academic background and justifying why it was a good tool, really, if that makes sense.***C1007** *They just wanted financial impact, how does it help them or how does it stop people coming into the clinic so much and how does it…? You know, they don’t like people not attending clinic when they’re booked in, they don’t like people not talking to them, not coming into clinic and they don’t like people having complications or needing to go to A&E and things like that. So if you talk about how it impacts on those key outcomes, then they’re interested.***C1011**

##### Intervention characteristics - the attraction, appropriateness, and power of network mapping

Intervention characteristics relate to the key features of an intervention which affect its implementation. In general terms, this social network intervention was viewed positively by all study participants. They considered it to be flexible, adaptable to different populations, and clearly differentiated from other resources. The strength of the underlying evidence base and its ability to bring about cost savings for health services were considered to promote its credibility and contributed to the perception of relative advantage over other available interventions.

Out of the three elements (network-mapping, needs assessment and linkage to local resources), the network-mapping component was considered to be the most useful, particularly the visualisation of an individual’s social networks. Network-mapping was perceived to provide a welcome opportunity for reflection and a structured conversation about social connectedness. Participants described how this articulation empowered users to start to prioritise their own needs, a useful first step in the process of promoting community integration.

Whilst the potential to access an up-to-date list of local community resources was valuable, participants raised a range of implementation challenges which detracted from this value. For example, the filtering capability of relevant resources was considered limited. The relevance and usefulness of suggested local support was dependent on resources being appropriately mapped, tagged and updated, which required significant investment of time and resources. This was particularly challenging given the fragile nature of funding for third sector organisations, with the results that the database infrastructure would likely become outdated relatively quickly. This could be off-putting for organisations when they were considering the possibility of mainstreaming GENIE™.

This issue of trustworthiness and confidence in this data was exacerbated by a range of technological problems and interface challenges when GENIE™ tried to access the database. Participants also identified a need for further filters and more advanced searching options. They raised safeguarding concerns about the lack of vetting of organisations, which could have future legal ramifications. Some previous users of GENIE™ felt that this issue could also lead to inactivity amongst those who use it, rendering any benefits unsustainable once contact with the intervention ceased.

As a result of these challenges with the final stage of the intervention, participants considered the third, resource linking aspect to be weak. The first (network-mapping) and second (needs assessment) elements of GENIE™ were considered to be the most usable and valuable in terms of bringing about therapeutic value for users.

##### Fit with approaches and characteristics of the outer setting

The ‘outer setting’ which comprises the characteristics of the external context in which an intervention is delivered impacting on implementation. GENIE™ was considered by participants to be closely aligned to the personalised care agenda and this, along with its solution-focused approach, was perceived to contribute to its value and suitability for implementation in mental health services.

Participants coalesced in their views that the intervention had clear relevance to the needs of mental health service users. However, they identified a range of barriers that might influence individual uptake of the intervention and prevent engagement with community activities. Poor provision of local infrastructure and the poverty of local environments created barriers for people. These barriers included a lack of transport, competing priorities, digital poverty, limited financial resources, childcare or caring responsibilities, and reduced capacity as a result of mental health symptoms.

Participants who had experience of facilitating GENIE™ also described challenges in engaging with people from diverse communities. Those more likely to be excluded were people with severe physical illness or disability, those who were very socially isolated and those from minority ethnic groups. As a result, participants felt that acceptable delivery of GENIE™ required a good understanding of the characteristics and needs of the target population, in order to ensure equity of access and benefit. Participants felt that organisations which were embedded in local communities and had high levels of social capital were best placed to deliver this type of social network intervention.

Financial pressures, extant cultures and limited resources within the statutory and voluntary sector were also identified as potential barriers to successful implementation. Participants felt that the impact of this could be mitigated by ensuring that the use of these types of interventions is closely aligned to existing systems, in order to minimise burden to organisations (e.g., by using GENIE™ as part of care planning approach or discharge planning).

##### Inner setting fit with existing organisation culture and work practices

The inner setting refers to the features of the internal context in which an intervention is delivered impacting on implementation. Participants highlighted the importance of GENIE™’s alignment to the culture and ethos of the implementing organisation as well as the intervention’s fit with existing systems. It was also considered important that the organisation workforce had the requisite skills and capacity to deliver the intervention and to maximise potential reach. There could be a tension between the willingness or desire to implement such an intervention and the capacity to deliver and reach potential beneficiaries.

Buy-in at all levels of an organisation, including managerial buy-in, was considered to be fundamental to successful implementation of GENIE™. Managerial buy-in increased the likelihood that protected time would be given to training for and delivery of the intervention, and that the intervention could be fully integrated into existing roles and systems. This process could also be supported by having champions within an organisation who could drive forward implementation.

Readiness to implement GENIE™ also requires IT support to facilitate intervention delivery, to ensure that the database is up-to-date, and to deal with any technical hitches. Facilitators should be given dongles to ensure internet access when delivering the intervention off-site and they should take paper copies of intervention materials as a back-up.

##### Characteristics of individuals: facilitators sensitivity to personal and social circumstances and the importance of rapport

Successful implementation of any intervention is heavily reliant on those delivering or facilitating it. Facilitation was identified as the key mechanism through which benefits of the intervention could be realised. This could be a complicated and delicate process which required significant skill, relational expertise, and confidence on the part of facilitators. It could be especially challenging when those accessing GENIE™ had limited support from social networks.

In light of this importance of skilled facilitation, participants emphasised key attributes and skills required in facilitators. These included sufficient time and resources to deliver GENIE™, a relevant professional background; appropriate personal values; adequate knowledge of the local area; an understanding of the value of the social aspects of health; empathy and rapport-building skills; and the ability to take a person-centred approach. These valued characteristics were felt by some participants to be especially prevalent in peer facilitators.

Participants also highlighted the importance of mental health specific training to support the delivery of GENIE™ in this context (see below).

##### Process of implementation – the need for buy-in, flexibility and adequate planning

Participants identified a range of potential impediments to the successful implementation of GENIE™. Dedicating sufficient time and resources to planning implementation was considered to be important. Set-up meetings were felt to be particularly effective for this, especially when attended by as many people within and around the organisation as possible. This was considered to promote transparency and to promote optimal allocation of the practical tasks associated with implementing the intervention.

Participants agreed that GENIE™ was better implemented and delivered face-to-face rather than remotely. This appeared to be due to the significant engagement work required which was considered best done in-person and the emphasis on the visual aspects of GENIE™. Reluctance to deliver GENIE™ remotely was heightened by online experiences during the recent covid-19 pandemic including zoom fatigue and challenges engaging with people remotely.

Flexibility in terms of supporting local and individual adaptations to the delivery of GENIE™ was considered to promote successful implementation. Participants believed that social connectedness interventions work best when personalised and tailored to the individual. This was felt to be particularly the case if potentially using GENIE™ with those with mental health difficulties, where flexibility in the timing of delivery was key. For example, some people would benefit most using GENIE™ on referral to a mental health service, while others would find it more helpful at discharge. Decisions needed to be made on a case-by-case basis and based on the needs and preferences of the individual. However, participants agreed that the intervention should not be used during acute periods of illness or as a way to hasten discharge from services.

Participants emphasised the importance of adequate and ongoing training to support the delivery^.^ This was seen as needing to include mental health specific GENIE™ training if using the intervention within a mental health context, and organisations should provide opportunities for facilitators to access reflective peer learning. Training was seen to need a focus on practical and engagement activities and how to take a personalised approach to delivery, with less attention given to the research or academic background of developing the intervention.

As part of the ongoing process of implementation, the benefits - both in terms of therapeutic benefit for the individual and organisational benefits such as cost savings - should be demonstrable both to facilitators and to members of the wider organisation. This was seen to promote ongoing buy-in and engagement.

### Phase 2

The consultation workshops demonstrated unanimous support across all stakeholder groups for the use of a social network intervention which had as its focus the facilitation of social integration and access to activities and connections to others in mental health services. Stakeholders did suggest a range of strategies and considerations for implementation. Participants felt that whilst in principle the ideas of community resource-mapping were of value in principle, in practice it would be challenging to implement and maintain and added complexity and burden to what was seen to work best which was the network mapping and eliciting preferences. Participants felt a broader national and online support, including advocacy groups and service user/carer forums would be of most use.

Participants coalesced in their views of where social network interventions might work best within secondary mental health services. These include:

- Care planning meetings,
- Discharge from hospitals,
- Early intervention services.

Participants also made detailed suggestions for ensuring acceptability to mental health service users. These included making participation in the intervention optional and accessible, providing opportunities for service users to invite a carer, friend or family member to take part in the facilitation of the intervention alongside them, and undertaking personalised approaches to facilitation with explicit acknowledgement that one approach is unlikely to work for everyone.

A full list of identified priorities for the future use of GENIE™, organised by stakeholder group, can be found in Table 4.

**Table 4:** Recommendations for implementation of GENIE™ in mental health services: findings from ConNEct Phase 2 consultation workshops

Recommendation	Service Users	Carers/Supporters	Professionals and Volunteers
GENIE™ should signpost to resources and support beyond the local area, e.g. national organisations and online resources.	x	x	x
There should be mechanisms for follow up after use of GENIE™: were people able to access what they were signposted to? If not, why not?	x		x
GENIE™ facilitation should factor in how people’s mental health will affect their ability to engage and where they may need extra support (low motivation, social anxiety, agoraphobia, medication side effects, etc).	x	x	
Facilitator training will be key: facilitators need to be compassionate, sensitive and highly-skilled. Incorporate practice and role-playing into the training.	x	x	x
Discharge from hospital or from secondary care would be a good time to use GENIE™.	x	x	x
When someone is transitioning from supported housing to independent living arrangements would be a good time to use GENIE™.	x		
GENIE™ would be valuable in a therapeutic art context.	x		
It would be beneficial to use GENIE™ repeatedly on a longer-term basis.	x	x	
GENIE™ would be a useful early intervention tool.	x	x	x
It would be beneficial to use GENIE™ as part of care planning and revisit it periodically with a care co-ordinator.	x		x
GENIE™ should factor in people’s varying mobility and access to transport. The facilitator will have a role in this but it also needs to be clear, for example, which venues are wheelchair-accessible and have transport links.	x	x	
Time and resource should be allocated to keeping GENIE™’s database up-to-date.		x	
GENIE™ facilitation should be flexible to accommodate the fact that the right time to use GENIE™ will be individual to each person depending on their individual circumstances.		x	x
Time and resource should be invested in making sure that all the people who might benefit from using GENIE™ have the opportunity to hear about it. This should include carers and factor in people’s differing communication needs and preferences. Suggested channels: leaflets, email, social media, Jobcentre Plus, local and community radio, TV, ORCHA, NHS website, healthcare settings.		x	
There should be provision of financial support for those who need this in order to increase accessibility and ensure the most people possible can benefit from GENIE™.		x	
GENIE™ should avoid overwhelming people with too much information.		x	
People should be offered the opportunity to invite a carer, friend or family member to join them for the sessions using GENIE™ with the facilitator.		x	
Time and resource should be allocated to building strong relationships with community and voluntary organisations. This will significantly enhance GENIE™’s accessibility to service users, the relevance and accuracy of the contents of the database, and successful implementation in services.		x	
Careful consideration is needed around which community spaces GENIE™ includes in its database: not all are positive or therapeutic. Some sort of vetting process may be needed. Carers could also play an important role.		x	x
GENIE™ should be available in community or voluntary mental health settings as well as NHS secondary care to promote accessibility and inclusivity.		x	
Social workers and peer support workers are well-placed to facilitate GENIE™.			x
GENIE™ fits in well with routine care planning activity and need not be framed as a whole extra activity.			x
Staff time will be a barrier to implementation and this needs to be planned for. Longer than usual appointments may be needed for facilitation of GENIE™.			x
Facilitator training should include the importance of getting accurate information from the beginning, going over it a couple of times to make sure, and getting feedback as you go.			x
GENIE™ facilitation may be enhanced by taking place in a group setting.			x
It would be beneficial to use GENIE™ in primary care while people are waiting for a mental health assessment.			x
Support from senior management and a good balance between evidencing GENIE™ and not adding pressure to staff will be key to successful trial and implementation.			x
GENIE™ should have an exportable output element and/or link to clinical systems.			x

## Discussion

The ConNEct study was designed to co-adapt, with service users, carers/supporters, clinicians and volunteers, an existing, evidence-based social network intervention for use in secondary mental health services. This is needed because research has shown that whilst people with severe and/or enduring mental health difficulties can confer benefit from diverse and supportive social networks, they are also found to have smaller social networks and networks of poorer quality and social connectiveness and activities are a needed component for recovery [9–11].

Service users consistently value holistic care, particularly discussions and interventions related to their social networks, but many feel these approaches are under-utilised and their importance underacknowledged in secondary mental health services [39]. This may reflect the current organisation of health services which is focussed on diagnosis, severity and deficits rather than locality and need.

Many health professionals remain disconnected from every day and community settings and their potential therapeutic value [39]. Developing effective ways to support professionals to deliver this personalised, recovery-oriented model of health and social care, which considers an individual within their social environment as a public health concern requires greater investment and focus.

Participants across both stages of the co-adaptation process highlighted a range of problems associated with the final resource-mapping stage of the GENIE™ intervention. Logistical challenges associated with the hosting and maintenance of an up-to-date database which incorporates sufficient breadth of resources to suit the range of potential users has been highlighted previously [26]. As a result, participants across both phases questioned the need for this final aspect, especially given the perceived therapeutic value of the first two stages (visualisation and needs assessment) when used on their own. These concerns were echoed by members of the patient and public advisory group.

One potential solution is to remove this final element and supplement stages 1 (social network visualisation) and 2 (needs assessment) with a focussed and personalised discussion planning how to improve social connectedness and engagement with valued activities. Persisting with a cumbersome and untrustworthy element compounded by technological failure is likely to undermine the elements which work well on their own merit. The individualised nature of this final planning stage was considered to be essential for mental health service users, who face multiple and diverse barriers to community engagement. This recommendation is supported by previous research which has demonstrated that a facilitated social network mapping exercise, focused on the broader social environment and sense of place as the key mechanism of engagement, can facilitate meaningful conversations about future social connectedness [24, 40]. This has been shown to translate to measurable benefits for service users and their families. Our systematic review demonstrated that provision of a financial stipend, in place of this local resource-mapping, may confer similar levels of benefit for participants [20]. This financial stipend was also considered to mitigate barriers to community engagement (e.g., access to transport, childcare, and activities with a cost attached).

Supporting flexibility in the delivery of a social network intervention was highlighted as fundamental to successful implementation both by those who had previously adopted the intervention and in our systematic reviews. Social network interventions were considered to work best when they were oriented to local context and personalised. This was echoed in the consultation workshops and ongoing co-adaptation work. Suggestions included making the use of GENIE™ or similar social network tools optional and widely available and accessible at any point, offering users the opportunity to invite a carer, friend or family member to participate in intervention delivery sessions; and ensuring flexibility in the timing of delivery. Using personal network mapping in mental health services is not new and the principles can be simply undertaken in drawing a map and does not need to be part of formal trademarked intervention for example.

The challenges faced when implementing a complex intervention into open systems can never be predicted in their entirety and successful implementation requires a degree of responsive flexibility [41]. Preparatory work for definitive evaluation as undertaken in the current study, underpinned by theories of implementation research, can mitigate potential impediments to implementation [34]. The co-creation of a systematic implementation strategy facilitates a consistent approach to evaluation which protects fidelity whilst adequately incorporating requisite levels of flexibility. Future evaluations should follow MRC guidelines for the evaluation of complex interventions and incorporate both randomised controlled trials and nested mixed methods process evaluations [29]. This would facilitate an examination of the underlying mechanisms of impact; enable the potential for intervention adaptation to be realised; and support the identification of minimum conditions required for patient and organisational benefit.

Participants in our study identified a range of skills and characteristics required for therapeutic facilitation of GENIE™. These were seen to be more important than any clinical or professional background. Individual attributes that were seen to be necessary included an empathetic approach, an understanding of the social aspects of health, relational skills, and knowledge of local communities. Furthermore, participants raised the importance of aligning facilitation with existing systems and processes within organisations, not least to mitigate against perceptions that delivering the intervention would increase workloads and be unmanageable for staff. This is particularly important given the financial and resource-related pressures that mental health services are currently facing [42]. Participants also reported the value of peer facilitation, which has also been highlighted in the previous implementation of interventions for both physical and mental health conditions [43–45]. However, evidence to support this approach for social network interventions for people with mental health difficulties was minimal within our systematic reviews and further research is required to understand the potential of peer facilitation in this context [20]. Particular attention should be given to development of job descriptions and personal specifications for facilitators and associated training and development plans. Participants also advocated for the development of mental health specific training for those facilitating these types of interventions in a mental health context, given the differences in the experience of mental and physical health conditions and additional barriers to engagement that mental health symptoms can create.

Our systematic reviews demonstrated the potential effectiveness of social network interventions for people with mental health difficulties but identified a lack of patient and public involvement in the extant evaluations of social network interventions across the world [20]. Future evaluations of these types of interventions should ensure adequate levels of PPI and draw on recent toolkits designed to promote meaningful and flexible involvement in clinical trials [46]. The full evaluation of GENIE™ in secondary mental health services will build upon and develop the PPI that has been so important to the project thus far. The work of the advisory group has already transcended an advisory role: we plan to formalise this by upgrading it to an involvement group whose remit explicitly encompasses active participation in all elements of the project, including design and oversight of the trial. The involvement group will coproduce materials and a support package for participants, and they will work with the research team to develop an agreed core outcome set and to gather, analyse and write up the resulting data. We aim to expand the group to 12 members and to introduce a targeted recruitment strategy to improve diversity, using the 9 protected characteristics of the Equality Act 2010 to ensure representation of a wider range of marginalised groups. This will ensure that the evaluative design is relevant and appropriate; that included measures reflect patient-prioritised outcomes; that trial processes are fit for purpose; that trial participants are meaningfully supported; and that acceptability to service users is prioritised, supporting recruitment and retention of trial participants and promoting successful implementation of the intervention in future.

Our study draws its strength from a combination of in-depth and collaborative research approaches and from the use of a recent implementation science framework to underpin study design. PPI was a particular strength: contributors brought their lived experience of mental health difficulties as co-researchers to the design of data collection tools, such as interview schedules and consultation workshops, and to data interpretation and analysis. This has enhanced the depth and nuance of our findings and will help to ensure that GENIE™ is developed and implemented to be truly beneficial to mental health service users. Data were collected in one site in the UK as this is expected to be where the intervention will be implemented and evaluated in the next stage of the programme of research. As such, an in-depth understanding of this implementation context will support the clinical trial and evaluation. Further research to implement such interventions should consider similar preparation and planning in order to fully understand local contextual factors that could impact intervention uptake and use in order to support equality, diversity and inclusion in future use and evaluation of such interventions [47, 48].

## Conclusion

We have identified a pressing need to implement social network interventions within mental health services. Our systematic reviews highlight the potential effectiveness of these types of interventions but identify a lack of rigorous and user-centred evaluations. Patient and public involvement is also lacking across this field of study, and its importance in every phase of both research and implementation has been another of our key findings. Our study has derived significant value from PPI across all study elements.

The systematic reviews and empirical work identified a range of factors that must be considered for successful implementation of interventions such as GENIE™. We have synthesised this information and co-produced an evidence-based implementation strategy and evaluation protocol. The next planned phase of the co-produced programme of work is to design and implement a randomised controlled trial to evaluate its clinical and cost effectiveness within secondary mental health services.

## Supplementary Information

**Additional file 1:** **Appendix 1.** Additional information on the GENIE™ intervention (taken from James E, Kennedy A, Vassilev I, Ellis J, Rogers A. Mediating engagement in a social network intervention for people living with a long-term condition: A qualitative study of the role of facilitation. Health Expect. 2020;23(3):681-690. doi:10.1111/hex.13048).

**Additional file 2:** **Appendix 2.** Example interview questions.

## Data Availability

The datasets generated and/or analysed during the current study are not publicly available due to ethical restrictions but are available from the corresponding author on reasonable request.

## Abbreviations

CFIR: Consolidated Framework for Implementation Research
GENIE™: Generating Engagement in Networks Involvement intervention
NHS: National Health Service
MMAT: Mixed methods appraisal tool
PPI: Patient and Public Involvement
RCT: Randomised controlled trial
UK: United Kingdom
USA: United States of America
