# Supplementary material for: Adapting a social network intervention for use in secondary mental health services using a collaborative approach with service users, carers/supporters and health professionals in the United Kingdom
Source: BMC Health Serv Res. 2022 Sep 9;22:1140. doi: 10.1186/s12913-022-08521-1 (PMC9461266; doi:10.1186/s12913-022-08521-1)
Supplement: Supplementary file 1 — Additional file 1: Appendix 1. Additional information on the GENIE™ intervention (taken from James E, Kennedy A, Vassilev I, Ellis J, Rogers A. Mediating engagement in a social network intervention for people living with a long-term condition: A qualitative study of the role of facilitation. Health Expect. 2020;23(3):681-690. doi:10.1111/hex.13048). [file 12913_2022_8521_MOESM1_ESM.docx]

| Step 1: Mapping using concentric circles | People, places, pets and objects (social network members) are mapped onto 3 concentric circles. Different circles capture the importance of each network member in supporting long-term condition (LTC) management, together with relationship and frequency of contact | - To explore how network members, contribute to SMS in everyday life - To create a visual image of existing support network - To guide a conversation about extending current support and accessing new sources of support - To capture change over time |
| --- | --- | --- |
| Step 2: Preference questionnaire | 13 online questions covering a range of local community activities and resources within the person's post-code area | - To find out what an individual enjoys doing or used to enjoy doing in the past - To link relevant network members to chosen activities - To select 3 most important activities in order to prevent information overload |
| Step 3: Linking to local activities | Intervention software selects from internal database all relevant local resources that correspond to individual's chosen activities. Information is displayed, including location of activity on Google Map |  |
