# Supplementary material for: Adapting a social network intervention for use in secondary mental health services using a collaborative approach with service users, carers/supporters and health professionals in the United Kingdom
Source: BMC Health Serv Res. 2022 Sep 9;22:1140. doi: 10.1186/s12913-022-08521-1 (PMC9461266; doi:10.1186/s12913-022-08521-1)
Supplement: Supplementary file 2 — Additional file 2: Appendix 2. Example interview questions. [file 12913_2022_8521_MOESM2_ESM.docx]

Appendix 2: Example interview questions

- BRIEF DESCRIPTION OF CURRENT ROLE
- DESCRIPTION OF EXPERIENCE OF IMPLEMENTING SOCIAL NETWORK INTERVENTIONS.
- WHAT TYPES OF SOCIAL NETWORK INTERVENTIONS HAVE YOU USED BEFORE/WHEN/IN WHICH SETTINGS/WITH WHOM?
- EXPLORE IMPLEMENTATION USING THE FIVE CFIR COMPONENTS:
  - *Intervention characteristics*: aspects of an intervention that may impact implementation success, including its perceived internal or external origin, evidence quality and strength, relative advantage, adaptability, trialability, complexity, design quality and presentation, and cost.
  - *Outer setting*: external influences on intervention implementation including patient needs and resources, cosmopolitanism or the level at which the implementing organization is networked with other organizations, peer pressure, and external policies and incentives.
  - *Inner setting*: characteristics of the implementing organization such as team culture, compatibility and relative priority of the intervention, structures for goal‐setting and feedback, leadership engagement, and the implementation climate.
  - *Characteristics of individuals*: individuals' beliefs, knowledge, self‐efficacy, and personal attributes that may affect implementation.
  - *Process of implementation*: stages of implementation such as planning, executing, reflecting and evaluating, and the presence of key intervention stakeholders and influencers including opinion leaders, stakeholder engagement, and project champions.
- WHAT WAS THE BIGGEST FACILITATOR/BARRIER TO IMPLEMENTATION?
- EXPLORE LESSONS LEARNT AND WHAT THEY WOULD DO DIFFERENTLY IN THE FUTURE
- WHO DO SOCIAL NETWORK INTERVENTIONS WORK BEST WITH?
  - WHY
- WHO DO SOCIAL NETWORK INTERVENTION NOT WORK WELL WITH?
  - WHY
- WHAT SUGGESTIONS WOULD YOU MAKE TO SOMEONE USING GENIE IN MENTAL HEALTH SERVICES?
